# 广西地区非小细胞肺癌中*K-ras*基因点突变的研究

**DOI:** 10.3779/j.issn.1009-3419.2011.06.06

**Published:** 2011-06-20

**Authors:** 炜祥 钟, 铭伍 陈, 磊 冼, 满红 李

**Affiliations:** 530021 南宁，广西医科大学第一附属医院心胸外科 Department of Cardiothoracic Surgery, the First Afliated Hospital of Guangxi Medical University, Nanning 530021, China

**Keywords:** 肺肿瘤, *K-ras*基因, 点突变, Lung neoplasms, *K-ras* gene, Point mutation

## Abstract

**背景与目的:**

近期研究显示存在*K-ras*基因突变的非小细胞肺癌（non-small cell lung cancer, NSCLC）患者对表皮生长因子受体-酪氨酸激酶抑制剂（epidermal growth factor receptor-tyrosine kinase inhibitors, EGFR-TKIs）耐药。本研究检测广西地区NSCLC中*K-ras*基因密码子12、13及61点突变，旨在探讨*K-ras*基因突变与广西地区NSCLC的关系。

**方法:**

采用聚合酶链反应-单链构象多态性（polymerase chain reaction-single-strand conformation polymorphism, PCR-SSCP）分析法与PCR-DNA序列分析法联合检测105例新鲜NSCLC手术切除标本和30例癌旁正常肺组织中*K-ras*基因密码子12、13及61点突变。

**结果:**

105例NSCLC癌组织及30例癌旁正常肺组织中无1例发生*K-ras*基因点突变，本研究选取的NSCLC病例组的*K-ras*基因突变率为0（0/105）。

**结论:**

广西地区NSCLC中*K-ras*基因野生型的比例高，提示广西地区NSCLC患者更能从EGFR-TKIs治疗中获益。

## Abstract (en)

**Background and objective:**

Recent studies indicated that non-small cell lung cancer (NSCLC) patients with mutant *K-ras* were resistant to epidermal growth factor receptor-tyrosine kinase inhibitors (EGFR-TKIs). The aim of this study is to explore the relationship between the mutation of *K-ras* gene and NSCLC in Guangxi by detecting the point mutations in codon 12, 13 and 61 of *K-ras* gene in NSCLC.

**Methods:**

The point mutations in codon 12, 13 and 61 of *K-ras* gene were detected by single-strand conformation polymorphism (SSCP) analysis of polymerase chain reaction (PCR) products and DNA sequencing analysis in 105 cases of NSCLC tissues and 30 cases of adjacent normal tissues.

**Results:**

No point mutation in codon 12, 13 and 61 of *K-ras* gene was found in 105 cases of NSCLC tissues and 30 cases of adjacent normal tissues. In this study, the mutation frequency of *K-ras* gene in NSCLC was 0 (0/105).

**Conclusion:**

The high proportion of *K-ras* gene in wild-type indicates that patients with NSCLC in Guangxi could take more benefits from the therapy with EGFR-TKIs.

*K-ras*基因是肺“启动”癌变的关键基因之一^[1]^。现已证实*K-ras*基因与肺癌的发生、发展甚至是预后均有着密切的关系。流行病学研究^[2]^表明肺癌的发病具有地域分布与种族差异。目前国内各地关于*K-ras*基因点突变与肺癌的研究不少，但针对广西地区的罕见。本研究应用聚合酶链反应-单链构象多态性（polymerase chain reaction-single-strand conformation polymorphism, PCR-SSCP）分析法与PCR-DNA序列分析法联合检测非小细胞肺癌（non-small cell lung cancer, NSCLC）中*K-ras*基因密码子12、13及61的点突变，旨在探讨*K-ras*基因突变与广西地区NSCLC的关系。

## 1 材料与方法

### 1.1 材料

#### 1.1.1 标本来源

收集广西医科大学第一附属医院心胸外科2007年9月-2010年5月手术切除的105例新鲜NSCLC标本，其中男性69例（65.7%），女性36例（34.3%）；汉族77例（73.3%），壮族24例（22.9%），瑶族2例（1.9%），毛南族2例（1.9%）；年龄范围24岁-84岁，中位年龄为55岁；无吸烟史57例（54.3%），有吸烟史48例（45.7%）。所有病例均由术后组织病理学诊断证实。按肺肿瘤分类2004（WHO）标准进行病理分型，鳞癌23例（21.9%），腺癌69例（65.7%），其中细支气管肺泡癌15例（14.3%）；腺鳞癌6例（5.7%）；其它类型7例（6.7%），包括大细胞癌4例（3.8%），不典型类癌1例（1.0%），粘液表皮样癌2例（1.9%）。按国际抗癌联盟标准（1997）进行临床分期，Ⅰ期、Ⅱ期58例（55.2%），Ⅲ期、Ⅳ期47例（44.8%）。肿瘤细胞分化程度好（分化级别为Ⅰ+Ⅰ-Ⅱ+Ⅱ级）71例（67.6%），分化程度差（分化级别为Ⅱ-Ⅲ+Ⅲ+Ⅳ级）34例（32.4%）。病理证实无淋巴结转移63例（60.0%），有淋巴结转移42例（40.0%）。所有患者均为初治，术前未行放、化疗或靶向治疗。同时取其中30例距癌灶边缘5 cm以外并经术后病理检查证实无癌细胞浸润的正常肺组织做对照，其中男性23例（76.7%），女性7例（23.3%），年龄范围39岁-74岁，中位年龄为52岁。

#### 1.1.2 引物的设计与合成

各引物序列参考文献^[3]^，由北京三博远志生物技术有限责任公司合成。

### 1.2 方法

#### 1.2.1 DNA的提取及纯度

应用DNA提取试剂盒（离心柱型）提取组织中DNA，经紫外分光光度计作DNA纯度检测。

#### 1.2.2 PCR扩增

反应体系为50 μL，包括：双蒸水（double distilled water, ddH_2_O）21.0 μL、引物上、下游（10 μmol/ L）各1.0 μL、模板DNA 2.0 μL及2×Taq PCR Master Mix 25.0 μL；反应条件：95 ℃ 3 min，95 ℃ 30 s、退火温度（an-nealing temperature, Ta）30 s、72 ℃ 45 s进行X个循环（其中，K-ras-exon 2的Ta为54.5 ℃，X为30；K-ras-exon 3的Ta为56.5 ℃，X为35），72 ℃ 5 min。

#### 1.2.3 SSCP分析

取2 μL PCR产物与2 μL变性上样液（98%去离子甲酰胺、10 mmol/L乙二胺四乙酸、0.025%溴酚蓝及0.025%二甲苯氰）混匀，PCR仪中98 ℃变性10 min后立即置于冰上，5 min-10 min后取2.5 μL上样，在12%的聚丙烯酰胺凝胶（Polyacrylamide gel, PAG）[其总体积为50 mL，配方：ddH_2_O 19.7 mL、30%丙烯酰胺混合液（丙烯酰胺：甲叉双丙烯酰胺为29:1）20 mL、5×TBE 10 mL、10%过硫酸铵350 μL及四甲基乙二胺33 μL]中180 V（即10 v/cm）电泳。当溴酚蓝接近凝胶下缘（一般为5.5 h-6.0 h）时取下凝胶，采用银染法染色：10%乙醇固定5 min；1%硝酸氧化3 min，ddH_2_O淋洗1 min；0.2%硝酸银染色25 min-30 min，ddH_2_O淋洗1 min；3%碳酸钠+0.019%甲醛显色至条带清晰；10%冰乙酸终止显色2 min，ddH_2_O淋洗1 min。最后予保鲜膜覆盖、拍照、分析并记录结果。SSCP结果判定：以等位条带泳动异常或多出泳动条带初步判断存在点突变，筛选出来的样本进一步予DNA序列分析确认。

#### 1.2.4 DNA序列分析

取20 μL PCR产物送予北京诺赛基因组研究中心有限公司进行测序。其中，K-ras-exon 2 PCR产物从正、反两个方向测序，K-ras-exon 3 PCR产物从反向测序。

### 1.3 统计学分析

本组资料为计数资料，以百分数表示。应用SPSS 13.0软件，采用χ^2^检验或*Fisher*确切概率检验进行统计学分析，以*P* < 0.05为差异有统计学意义。

## 2 结果

### 2.1 PCR扩增

以K-ras-exon 2/3引物特异性扩增NSCLC癌组织及癌旁正常肺组织，获得115 bp的K-ras-exon 2片段与131 bp的K-ras-exon 3片段（图 1）。经测序验证扩增产物与目的片段相符合。

**1:**
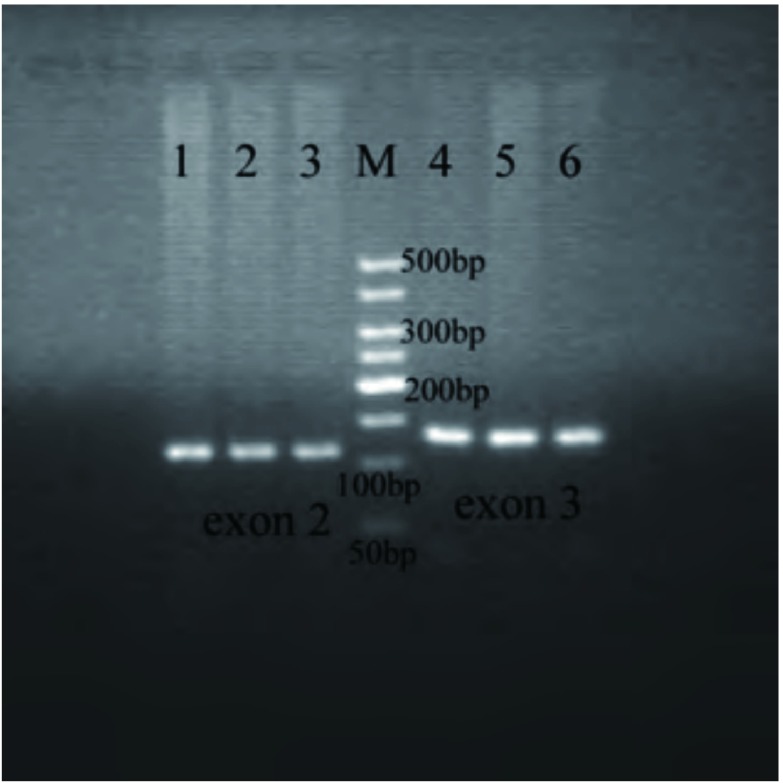
*K-ras*基因PCR扩增产物凝胶电泳成像图（6 cm:6 cm）。1-2：NSCLC癌组织；3：癌旁正常肺组织；M：50 bp DNA marker；4-5；NSCLC癌组织；6：癌旁正常肺组织。 Gel electrophresis photographs of PCR amplified products for *K-ras* gene (6 cm:6 cm). 1-3: K-ras-exon 2; 4-6 K-ras-exon 3; 1-2: NSCLC tissues; 3: adjacent normal tissues; M: 50 bp DNA marker; 4-5: NSCLC tissues; 6: adjacent normal tissues.

### 2.2 SSCP分析

在105例NSCLC癌组织及30例癌旁正常肺组织中，无1例*K-ras*基因突变（图 2，图 3），即本研究选取的NSCLC病例组的*K-ras*基因突变率为0（0/105）。期间曾筛选出9例疑似K-ras-exon 2突变的样本（其银染后在PAG上表现为两条ssDNA泳带之间多出一条泳带，如图 1箭头所示），经序列分析证实，均未见碱基突变。

**2:**
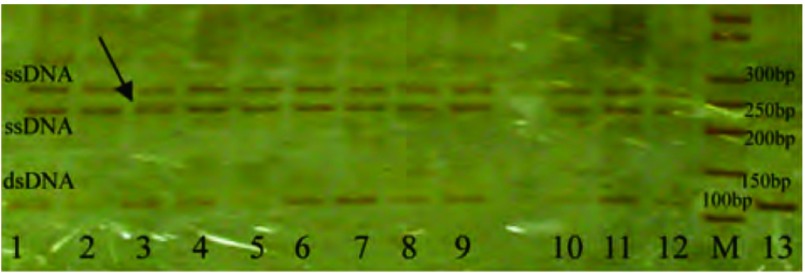
K-ras-exon 2 PCR产物的SSCP图像(3 cm:9 cm)。1-12：已变性的PCR产物；1-7：NSCLC癌组织；8-12：癌旁正常肺组织；M：50 bp DNA marker；13：未变性的NSCLC癌组织PCR产物，作为实验对照组。 the SSCP image of PCR products for K-ras-exon 2 (3 cm:9 cm). 1-12: denatured products of PCR; 1-7: NSCLC tissues; 8-12: adjacent normal tissues; M: 50 bp DNA marker; 13: undenatured product of PCR in NSCLC tissue, as a control group.

**3:**
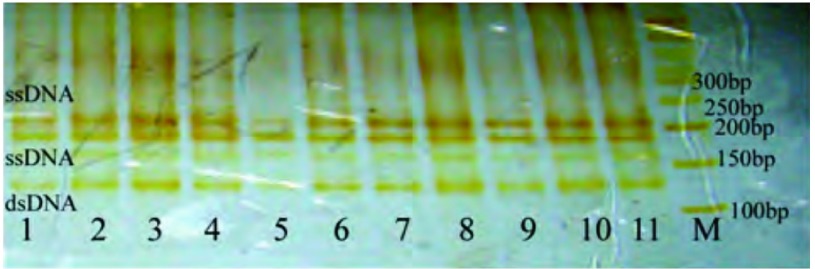
K-ras-exon 3 PCR产物的SSCP图像(3 cm:9 cm)。1-11：已变性的PCR产物；1-8：NSCLC癌组织；9-11：癌旁正常肺组织；M：50 bp DNA marker。 the SSCP image of PCR products for K-ras-exon 3 (3 cm:9 cm). 1-11: denatured products of PCR; 1-8: NSCLC tissues; 9-11: adjacent normal tissues; M: 50 bp DNA marker.

### 2.3 DNA序列分析

在105例NSCLC癌组织及30例癌旁正常肺组织中，无1例出现*K-ras*基因密码子12（GGT）或/和密码子13（GGC）或/和密码子61（CAA）中的碱基改变（图 4，图 5）。

**4:**
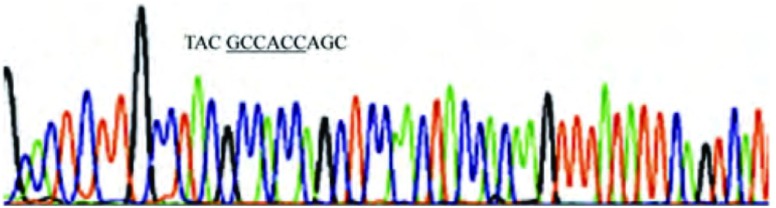
K-ras-exon 2 PCR产物的反向测序图(3 cm:9 cm)。*K-ras*基因密码子12、13均为野生型即GGT、GGC。 The reverse sequencing map of PCR products for K-ras-exon 2 (3 cm:9 cm). codon 12 of *K-ras* gene: GGT; codon 13 of *K-ras* gene: GGC.

**5:**
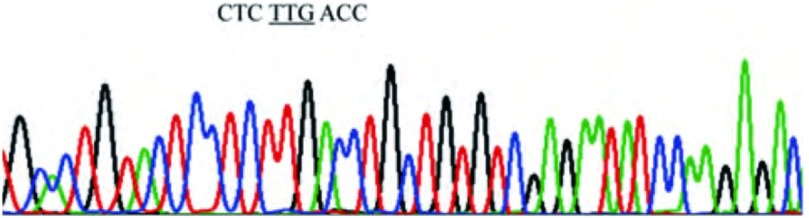
K-ras-exon 3 PCR产物的反向测序图(3 cm:9 cm)。*K-ras*基因密码子61为野生型即CAA。 The reverse sequencing map of PCR products for K-ras-exon 3 (3 cm:9 cm). codon 61 of *K-ras* gene: CAA.

## 3 讨论

*K-ras*基因属于RAS原癌基因家族，位于12号染色体上，主要功能是编码P21蛋白质（一种分子量为21 kDa的磷蛋白），后者位于细胞质膜内面，对GTP（guanosine triphosphate，三磷酸鸟苷）和GDP（guanosine diphos-phate，二磷酸鸟苷）均具有很强的亲和性，且具有同源性GTP酶活性，通过接受和传递细胞外生长刺激信号影响细胞内核酶等生物大分子合成，进而对细胞的生长和分化进行调控^[4]^。当*K-ras*基因Exon 2中的密码子12、13和Exon 3中的密码子61发生点突变而使*K-ras*基因被激活，可导致p21蛋白在关键位点（第12、13及61）发生氨基酸改变，引起K-ras蛋白的异常表达^[5]^，导致p21蛋白结构改变并一直处于激活状态，失去了其原有的信号传导功能，持续地激活磷脂酶并产生第二信使，造成细胞不可控制地增殖、恶变，最终导致肺癌的发生。

肺癌中*K-ras*基因点突变主要发生在NSCLC，常见的突变位点是*K-ras*基因Exon 2的密码子12、13及Exon 3的密码子61^[6]^。而NSCLC中近97%的*K-ras*基因突变涉及密码子12及13^[7]^。本研究应用PCR-SSCP分析法与PCR-DNA序列分析法联合检测了105例NSCLC癌组织及30例癌旁正常肺组织中*K-ras*基因密码子12、13及61的点突变，未发现有*K-ras*基因碱基改变，即本研究选取的NSCLC病例组的*K-ras*基因突变率为0（0/105）。此结果与西方国家约15%-30%的NSCLC患者存在*K-ras*基因突变的研究^[8, 9]^发现明显不相符合，与国内刘峰（2.9%）^[10]^、Li（5.8%）^[11]^、张阳（3.8%）^[12]^、及孙蕾娜（4.7%）^[12]^等学者报道的中国NSCLC患者*K-ras*基因突变情况亦有所不同。顾其华等^[14]^应用PCR和测序方法分析33例肺鳞癌和27例肺腺癌*K-ras*基因外显子2和3的突变，同样未发现有*K-ras*基因密码子12、13及61中的碱基改变。

考虑主要有以下两方面原因：一是*K-ras*基因在广西地区NSCLC中确实突变少或无突变，即*K-ras*基因突变存在区域异质性；二是实验中*K-ras*基因突变检测存在假阴性，其可能原因主要有：①标本取样不当，即正常组织过多而肿瘤组织过少，造成突变检测结果假阴性；②检测方法有待优化，目前检测基因点突变的方法较多，各有优缺点，本研究应用了诸多方法中的PCR-SSCP分析法与PCR-DNA序列分析法。PCR-SSCP分析法是根据DNA单链在非变性PAGE中，其迁移率除与链的长短有关外，更主要决定于单链所形成的二级空间构象，若DNA片段中碱基发生突变，将会引起单链DNA二级空间结构的改变，使SSCP凝胶上出现异常移动的泳带。PCR-SSCP分析法是一种快速、灵敏且有效检测基因点突变的方法，其突变检出率在50%-80%之间，但它不能识别突变位置，且当碱基转换或颠换不影响DNA构像时其检出率较低，如当G-T之间的颠换，其检出率为57%，而且突变位点周围序列的变化对SSCP分析结果影响非常小，检测存在假阴性。除实验条件的局限之外，少数突变不能检出可能是由于点突变引起的空间构象变化甚微、迁移率相差无几所致，尤其是点突变发生在扩增片段的两端时^[15]^。本实验中有9例样本出现异常泳带而序列分析证实无碱基突变，考虑是在某个温度、某种电泳缓冲液浓度或是一定配比的凝胶中，一个等位基因会有两种构象，在非变性凝胶中电泳就会有两条泳带^[16]^。PCR-DNA序列分析法即直接测序法，是国际上公认的基因突变检测方法。由各种突变检测技术检测到的突变最后都得由测序来确定突变类型及突变位置，而且测序法检测突变的效率达到100%。当然，直接测序法也不能被当成是突变检测的金标准，杂合突变、胶压缩、GC富集区的存在等问题使得很难通过一次测序获得精确的数据。综合分析，通过严格取材，联合应用PCR-SSCP分析法与PCR-DNA序列分析法，部分样本经二次、甚至多次测序确认等措施，本研究已将*K-ras*基因突变检测中存在的假阴性率控制到了最低。故考虑*K-ras*基因在广西地区NSCLC中突变少或无突变是由其区域异质性所致。

*K-ras*基因表达产物作为重要的表皮生长因子受体信号转导的下游效应子，其基因突变与患者对EGFR-TKIs耐药呈正相关，故对K-ras测序可能有助于选择患者接受TKI治疗^[17]^。本研究中，广西地区NSCLC中*K-ras*基因野生型的比例高，提示广西地区NSCLC患者更能从EGFR-TKIs治疗中获益。由于纳入研究的样本量相对较少，有待更多大样本进行进一步的研究。

## References

[1] Johnson L, Mercer K, Greenbaum D (2001). Somatic activation of the *K-ras* oncogene causes early onset lung cancer in mice. Nature.

[2] Wang J, Xu F, Zhou QH (2005). Advances in epidemiology of lung cancer. Chin J Lung Cancer.

[3] Lehman TA, Scott F, Seddon M (1996). Detection of *K-ras* oncogene mutations by polymerase chain reaction-based ligase chain reaction. Anal Biochem.

[4] Bos JL (1989). ras oncogenes in human cancer: a review. Cancer Res.

[5] Quinlan M P, Quatela SE, Philips MR (2008). Activated Kras, but not Hras or Nras, may initiate tumors of endodermal origin via stem cell expansion. Mol Cell Biol.

[6] Rodenhuis S, van de Wetering ML, Mooi W J (1987). Mutational activation of the *K-ras* oncogene. A possible pathogenetic factor in adenocarcinoma of the lung. N Engl J Med.

[7] Forbes S, Clements J, Dawson E (2006). COSMIC 2005. Br J Cancer.

[8] Massarelli E, Varella-Garcia M, Tang X (2007). *KRAS* mutation is an important predictor of resistance to therapy with epidermal growth factor receptor tyrosine kinase inhibitors in non-small-cell lung cancer. Clin Cancer Res.

[9] Marks JL, Broderick S, Zhou Q (2008). Prognostic and therapeutic implications of EGFR and KRAS mutations in resected lung adenocarcinoma. J Thorac Oncol.

[10] Liu F, Jiang B, Gong SJ (2007). Mutational analysis of *EGFR* and *K-RAS* in Chinese patients with non-small cell lung cancer. Chin J Med Genet.

[11] Li M, Liu L, Liu Z (2009). The status of *KRAS* mutations in patients with nonsmall cell lung cancers from mainland China. Oncol Rep.

[12] Zhang Y, Pan ZK, Zhang X (2010). *K-RAS* gene mutations in patients with nonsmall cell lung cancer. Chin J Lung Cancer.

[13] Sun LN, Luan HL, Zang FL (2010). Relationship between *EGFR* and *K-ras* mutations and clinicopathological characteristics and response to erlotinib treatment in 301 Chinese patients with non-small cell lung cancer. Chin J Oncol.

[14] Gu QH, Chen Q, Zhang YQ (2003). Sequence analysing of *Ki-ras* and *p53* gene in patents with lung squamous cancer and lung adenocarcinoma. Chin J Mod Med.

[15] Neri A, Baldini L, Trecca D (1993). *p53* gene mutations in multiple myeloma are associated with advanced forms of malignancy. Blood.

[16] Wang N, Lian YS, Liu YX (2008). Optimization of PCR-SSCP analysis method. Prog Mod Biomed.

[17] Finberg KE, Sequist LV, Joshi VA (2007). Mucinous differentiation correlates with absence of *EGFR* mutation and presence of *KRAS* mutation in lung adenocarcinomas with bronchioloalveolar features. J Mol Diagn.

